# Splicing factor SRSF3 represses translation of p21^cip1/waf1^ mRNA

**DOI:** 10.1038/s41419-022-05371-x

**Published:** 2022-11-07

**Authors:** Jeeho Kim, Ra Young Park, Younghoon Kee, Sunjoo Jeong, Takbum Ohn

**Affiliations:** 1 Department of Cellular & Molecular Medicine, College of Medicine, Chosun University, Gwangju, 61452 South Korea; 2 Laboratory of RNA Cell Biology, Department of Molecular Biology, Graduate Department of Bioconvergence Engineering, Dankook University, Jukjeon, Yongin, Gyeonggi 16890 South Korea; 3 Cancer Mutation Research Center, College of Medicine, Chosun University, Gwangju, 61452 South Korea; 4 Department of New Biology, Daegu Gyeongbuk Institute of Science and Technology (DGIST), Daegu, 42988 South Korea; 5 Present Address: Bio-IT Foundry Center of Chonnam National University, 77 Yongbong-ro Buk-Gu, Gwanju, 61186 South Korea

**Keywords:** Cell growth, Senescence, Oncogenes, Translation, RNA metabolism

## Abstract

Serine/arginine-rich splicing factor 3 (SRSF3) is an RNA binding protein that most often regulates gene expression at the splicing level. Although the role of SRSF3 in mRNA splicing in the nucleus is well known, its splicing-independent role outside of the nucleus is poorly understood. Here, we found that SRSF3 exerts a translational control of p21 mRNA. Depletion of SRSF3 induces cellular senescence and increases the expression of p21 independent of p53. Consistent with the expression patterns of SRSF3 and p21 mRNA in the TCGA database, SRSF3 knockdown increases the p21 mRNA level and its translation efficiency as well. SRSF3 physically associates with the 3′UTR region of p21 mRNA and the translational initiation factor, eIF4A1. Our study proposes a model in which SRSF3 regulates translation by interacting with eIF4A1 at the 3′UTR region of p21 mRNA. We also found that SRSF3 localizes to the cytoplasmic RNA granule along with eIF4A1, which may assist in translational repression therein. Thus, our results provide a new mode of regulation for p21 expression, a crucial regulator of the cell cycle and senescence, which occurs at the translational level and involves SRSF3.

## Introduction

Serine/arginine (SR)-rich protein is an RNA-binding protein known as an alternative splicing regulator that regulates several aspects of gene expression [1]. Altered expression of the SR proteins has been implicated in various human diseases, including cancer [2–4]. SRSF3 (Serine/arginine-rich splicing factor 3) is upregulated in various cancers, including breast, ovarian, retinoblastoma, gastric, hepatocellular, and colorectal cancer [5, 6]. Moreover, SRSF3 is essential for embryonic development [7, 8], as knockout of SRSF3 in mice results in death at the morula stage of the embryo, failing to form blastocysts [8]. SRSF3 deficiency inhibits cancer cell proliferation, migration, and invasion [9, 10]. Thus, SRSF3 is essential for cell survival and development. Treatment with caffeine, digoxin, amiodarone, theophylline, and amiloride promotes G2/M arrest, senescence, and apoptosis in cancer cells [11, 12]. Moreover, these agents reduce SRSF3 [11, 13].

SR proteins are mainly located in the nucleus, reflecting their primary role as splicing factors [14–17]. However, studies found that some SR protein family members can shuttle between the nucleus and cytoplasm [15, 18, 19]. Specifically, SRSF1, SRSF3, and SRSF7 migrate to the cytoplasm and are involved in translation control [10, 20, 21]. For example, SRSF1 binds to the AU-rich elements (ARE) of the 3’UTR and inhibits mRNA translation [22]. Conversely, it interacts with mTOR to promote translation when it binds to the exonic splicing enhancer (ESE) of the coding region [20]. Similarly, SRSF3 regulates the translation of picornavirus mRNA by binding to PCBP2, an internal ribosome entry site (IRES) binding protein [23]. Moreover, SRSF3 binds to the 5′-UTR of PDCD4 and localizes PDCD4 mRNA to the P-body (PB) to inhibit translation [10]. In addition, SRSF3 is reported to be involved in the translation of inflammatory genes such as LCN2, CCL5, and CCL3 [24].

Uncontrolled cell proliferation is a hallmark of carcinogenesis, and cellular senescence is a state of permanent cell cycle arrest, and major changes in metabolic activity and cell morphology [25, 26]. Cellular senescence was originally defined as a permanent arrest in the G1 phase of the cell cycle [27, 28]. However, it was recently found that an arrest in the G2 phase induces cellular senescence [29, 30]. A crucial factor that controls the cell cycle and cellular senescence, p21^cip1/waf1^ (referred to as p21 hereafter), acts by inhibiting cyclin-dependent kinases (CDKs), including the CDK4/6-cyclin D complex and the CDK1-cyclin B complex. The induction of p21 is primarily under the control of p53-dependent transcription in response to cellular stress, such as DNA damage [31]. Moreover, p21 disrupts reactivation of the CDK-cyclin complex by cdc25, and overexpression of p21 induces cell cycle arrest and cellular senescence [32, 33]. As such, the p53-dependent activation of p21 is a key axis for progression through G1-S-G2 phases and dictates overall cellular proliferation [34]. Although many factors or mechanisms regulating p21 expression have been identified, the full repertoire of p21-controlling mechanisms has yet to be identified.

While analyzing the TCGA-Colon (COAD and COADREAD) database, we found a negative correlation between SRSF3 and p21 expression. We found that depletion of SRSF3 caused p21 upregulation independent of p53. We found that SRSF3 downregulated the translation of p21 by binding to the 3′UTR region, possibly by inhibiting eIF4A1. Elucidation of the mechanism of p21 regulation by SRSF3 will provide important clues for cancer treatment strategies.

## Materials and methods

### Cell culture and transfection

SW480, HT-29, HCT116 (human colon cancer cells), U2OS (human osteosarcoma), and HEK293T (human embryonic kidney) cells were grown in Dulbecco’s modified Eagle’s medium (Invitrogen, Carlsbad, CA, USA). All cell lines were purchased from American Type Culture Collection (ATCC, Manassas, VA, USA). All media were supplemented with 10% fetal bovine serum (FBS) and 1% penicillin/streptomycin antibiotic solution. Cells were maintained in 5% CO2 in a humidified atmosphere at 37 °C. Plasmids were transiently transfected into mammalian cells using TurboFect (Thermo Scientific, Waltham, MA, USA) or Lipofectamine 2000 (Invitrogen). After 36 h, cells were lysed using RIPA buffer (150 mM sodium chloride, 1% NP-40, 0.5% sodium deoxycholate, 0.1% SDS, and 50 mM Tris-HCL pH 8.0).

### Antibodies and reagents

The anti-SRSF3 (7B4) (sc-13510), anti-p21cip1/waf1 (C-19) (sc-397), anti-eIF3b (N-20) (sc-16377), anti-p53(DO-1) (sc-126), and anti-eIF4G (H-300) (sc-11373) antibodies were from Santa Cruz (Dallas, TX, USA), anti-FLAG (M2) antibody was from Sigma-Aldrich, anti-β-actin (AC-15) (ab6276), and anti-eIF4A1 (ab31217) antibodies were from Abcam (Cambridge, MA, USA); anti-rpS3 (#2579) and anti-rpL13a (#2765) antibodies were from Cell Signaling (Danvers, MA, USA), and anti-PARP (YF-MA10018) antibodies were from Abfrontier (Seoul, South Korea). Cycloheximide was purchased from Sigma-Aldrich (St. Louis, MO, USA).

### Plasmid constructs and cloning

Human SRSF3 cDNA was amplified from HEK293T cells by RT-PCR using the following primers: forward, 5′-AAAAGAATTCAATGCATCGTGATTCCTGTCCAT-3′ and reverse, 5′-AAAAGCGGCCGCCTATTTCCTTTCATTTGACCTAGATC -3′ and cloned into the pCI-neo-Flag mammalian expression vector (Promega, Madison, WI, USA). To prepare serial deletion constructs of SRSF3 (RRM [1-83], RS [84-164]), each fragment was PCR-amplified using pCI-neo-Flag-SRSF3 as a template, and the PCR products were inserted into the EcoRI and NotI sites of the pCI-neo-Flag vector and pGEX4T-3 vector. All the constructs were verified by sequencing. A comprehensive list of all the PCR primers used in this study is provided in Supplemental Table S3.

### RNA interference

The cells were transfected with siRNAs (40 nM) using Lipofectamine 2000 (Invitrogen). After 36 h, the cells were trypsinized, replated, and transfected again for another 36 h. The knockdown efficiency was verified by western blot analysis. The siRNA sequences used in this study are listed in Supplementary Table S4.

### Immunoprecipitation and western blot analysis

Cell extracts were prepared in cytoplasm extract buffer (20 mM HEPES (pH 7.6), 10 mM KCl, 1 mM EDTA, 0.2% NP-40, 10% glycerol, 1 mM DTT) containing protease inhibitors (1 mM Na2VO4, 10 mM NaF, 2 mM PMSF, 5 μg/ml leupeptin, 10 μg/ml aprotinin, and 1 μg/ml pepstatin A) (Roche, Switzerland). After incubating for 20 min, the samples were centrifuged at 2400 × *g* for 5 min. For immunoprecipitation of protein complexes, cell extracts were pre-cleared with protein G-Sepharose beads (GE Healthcare) and incubated with the appropriate antibodies. Immune complexes were then analyzed by immunoblotting, which was performed using antibodies. Equal amounts of proteins were separated by SDS-PAGE followed by electrotransfer onto PVDF membranes (PALL Life Sciences, USA), incubation with appropriate primary antibodies overnight at 4 °C, followed by incubation with peroxidase-conjugated secondary antibodies for 1 h at room temperature. The bands were visualized using an ECL chemiluminescence detection system (iNtRON Biotechnology, Korea).

### Ribonucleic acid-protein-immunoprecipitation (RNP-IP)

This method has been previously described [35]. Cells were washed in cold PBS and resuspended in cytoplasm extract buffer (20 mM Hepes (pH 7.6), 10 mM KCl, 1 mM EDTA, 0.2% NP-40, 10% glycerol, 1 mM DTT containing protease inhibitors (1 mM Na2VO4, 10 mM NaF, 2 mM PMSF, 5 μg/ml leupeptin, 10 μg/ml aprotinin, 1 μg/ml pepstatin A) (Roche, Indianapolis, IN, USA), 40 U/ml RiboLock RNase Inhibitor (Fermentas, Pittsburgh, PA, USA) in DEPC water)). After incubating for 20 min, the samples were centrifuged at 2400 × *g* for 5 min. The cytoplasmic extracts were immunoprecipitated with IgG, anti-SRSF3, or anti-Flag (M2) at 4 °C overnight. Beads were washed with cytoplasm extract buffer five times and extracted in Trizol. The RNA samples were treated with DNase (TURBO DNA-free kit) and used for RT-PCR.

### Quantitative PCR and RT-PCR

Total RNA was extracted from cell lysates using TRIzol (Invitrogen), and 2 µg of total RNA was reverse transcribed to cDNA using an oligo dT primer (Bioneer, Daejeon, Korea) and M-MuLV Reverse Transcriptase (Invitrogen). Real-time PCR analysis was performed using specific primers and the SYBR Premix Ex Taq™ kit (TaKaRa Bio, Shiga, Japan). Transcripts were detected using the CFX96 Real-Time PCR Detection System (Bio-Rad, Hercules, CA, USA). Each sample was analyzed in triplicate and the target genes were normalized relative to the reference housekeeping gene GAPDH. Relative mRNA expression levels were calculated using the comparative threshold cycle (Ct) method with GAPDH as the control, according to the following formula: ΔCt = Ct (GAPDH) − Ct (target gene). The fold change in gene expression normalized to GAPDH and relative to the control sample. It was calculated as 2−ΔΔ Ct and the qRT-PCR primer sequences are listed in Supplementary Table S5.

### Immunofluorescence analysis

Cells were grown on glass coverslips, washed twice in PBS-A (1× PBS with 0.02% sodium azide), immediately fixed with 4% (v/v) formaldehyde in PBS for 15 min at 4 °C, and permeabilized with 0.5% (v/v) Triton X-100 in PBS for 15 min at RT. Proteins were detected using a primary antibody overnight at 4 °C, then washed twice in PBS-A, and briefly stained with fluorophore-tagged (cy2 or cy3) secondary antibodies (Jackson ImmunoResearch, West Grove, PA, USA). Nuclei were counterstained with DAPI or Hoechst33258 (Sigma-Aldrich). Immunofluorescence was performed using a fluorescence microscope (Nikon, Tokyo, Japan).

### Polysome profiling analysis

Cell lysates of SW480 were prepared in polysome profiling buffer (20 mM HEPES (pH7.6), 125 mM KCl, 5 mM MgCl2, 2 mM DTT, and DEPC water) for sucrose gradient centrifugation. Extracts were incubated on ice for 15 min, and the insoluble material was pelleted by centrifugation at 13000 rpm for 15 min. The resulting supernatant extracts were then loaded onto a 17.5–50% sucrose gradient prepared with polysome profiling buffer and ultra-centrifuged for 2.4 h at 35,000 rpm in an SW41-Ti rotor (Beckman, Brea, CA, USA). Post centrifugation, the gradients were fractionated using a fraction collector (Brandel, Gaithersburg, MD, USA), and their quality was monitored at 253 nm using a UA-6 absorbance detector (Isco, Lincoln, NE, USA). Total RNA was extracted from each fraction using TriZol.

### Flow cytometry

The cells were washed twice with cold PBS and fixed with ice-cold 70% ethanol. Cells were incubated at −20 °C in a fixative for 2 h. The cells were centrifuged for 2 min at 5000 rpm and ethanol was aspirated. The cell pellets were washed with 1× PBS, resuspended in propidium iodide (PI)-staining solution (3 µg/ml PI, 0.5 mg/ml RNase A, and Triton X-100 in 1×PBS), and incubated at RT for 30 min before flow cytometric analysis (FACS Calibur, BD, San Jose, CA, USA).

### Luciferase reporter assay

Cells seeded in 12 well plates were transiently cotransfected with firefly luciferase reporter (50 ng), Renilla luciferase reporter (5 ng), and siRNA (40 nM). Luciferase activity was determined with a dual-luciferase assay system (Promega, Madison, WI, USA). The activity was determined using a Glomax 20/20 luminometer (Promega).

### Recombinant protein purification

To synthesize recombinant GST-SRSF3 full-length and its domains (RRM and RS) proteins, overnight cultures of E. coli were grown at 37 °C in LB medium supplemented with antibiotics (10 μg/ml ampicillin). To induce protein expression, it was supplemented with 0.25 mM IPTG and the cultures were incubated at 30 °C for an additional 3 h. E. coli pellets were resuspended in 3 ml of lysis buffer (1 mg/ml lysozyme, 50 mM Tris-HCl (pH7.6), 150 mM NaCl, 0.5% NP-40, 1 mM EDTA) and cells were lysed by sonication. To separate cell debris from the soluble supernatant, the suspension was centrifuged at (15,000 rpm for 15 min and 4 °C). The suspension was bound to glutathione-Sepharose beads overnight. After washing 4 times, it was eluted using an elution buffer (50 mM Tris-HCl (pH 8.0), 10 mM reduced glutathione, 150 mM NaCl). To synthesize the recombinant His_x6_ eIF4A1 protein, overnight cultures of E. coli were grown at 37 °C in LB medium supplemented with antibiotics (100 μg/ml kanamycin). To induce protein expression, supplemented with 0.4 mM IPTG and incubated the culture at 30 °C for an additional 4 h. E. coli pellets were resuspended in 20 ml of lysis buffer (150 mM NaCl, 50 mM Tris-HCl, 3 mM β-mercaptoethanol, 0.1 mM PMSF, pH 8.0) and cells were lysed by sonication. To separate cell debris from the soluble supernatant, the suspension was centrifuged (16,000 × *g* for 25 min and 4 °C). The suspension was bound to Nickel Sepharose beads. After washing, it was eluted using an elution buffer (150 mM NaCl, 50 mM Tris-HCl, 250 mM imidazole, 3 mM BME, pH 8.0).

### In vitro GST-pulldown assay

Bacterially expressed GST-fusion SRSF3 full length and its domains (RRM and RS) was immobilized onto Glutathione Sepharose 4B beads (GE Healthcare) and incubated with bacterially expressed His_x6_-eIF4A1 fusion protein, overnight at 4 °C. The GST bead-bound complexes were then washed five times with GST lysis buffer (20 mM HEPES (pH 7.6), 150 mM NaCl, 5 mM MgCl2, 1% Triton X-100, and 5% Glycerol), and bound proteins were separated by SDS-PAGE and analyzed by western blotting using appropriate antibodies.

### SA-β-gal staining

SW480, HCT116 (p53+/+) and HCT116 (p53−/−) cells were cultured in 6 well sterile culture plates and transfected with siControl or siSRSF3 for 72 h. Cells were stained with senescence-associated (SA) β-galactosidase staining kit (#9860, Cell signaling), according to manufacture’s protocol. The percentage of β-gal positive cells were the number of senescent cells divided by the total number of cells counted.

### Bioinformatics analysis of RNA-Seq data in TCGA

The Cancer Genome Atlas (TCGA; https://www.cancer.gov/about-nci/organization/ccg/research/structural-genomics/tcga) databases were downloaded using the UCSC Xena browser Data Hub (https://xenabrowser.net/hub/). RNA sequencing data as measured by Illumina HiSeq (RSEM normalized) were downloaded whenever available. The TCGA mRNA expression of discovery set was transformed into log^2^ scale. *P*-values between groups were calculated Welch’s corrected *t*-test unless otherwise mentioned using GraphPad Prism (GraphPad Software Inc., CA, USA). The number of patient samples used for TCGA analysis was colon (*n* = 492), esophageal (*n* = 197), lung (*n* = 1231), stomach (*n* = 417), head and neck (*n* = 566), pancreatic (*n* = 184), uterine (*n* = 201), kidney (*n* = 321).

### Statistics

Data are presented as the mean ± SEM of three independent experiments, and significant differences between groups were assessed by a two-tailed paired Student’s *t*-test or two-way ANOVA using GraphPad Prism (GraphPad Software Inc., CA, USA). Results with values of *P < 0.05, **P < 0.01, and ***P < 0.001 were considered statistically significant.

## Results

### Deficiency of SRSF3 leads to cellular senescence and upregulation of p21^cip1/waf1^

**Fig. 1:**
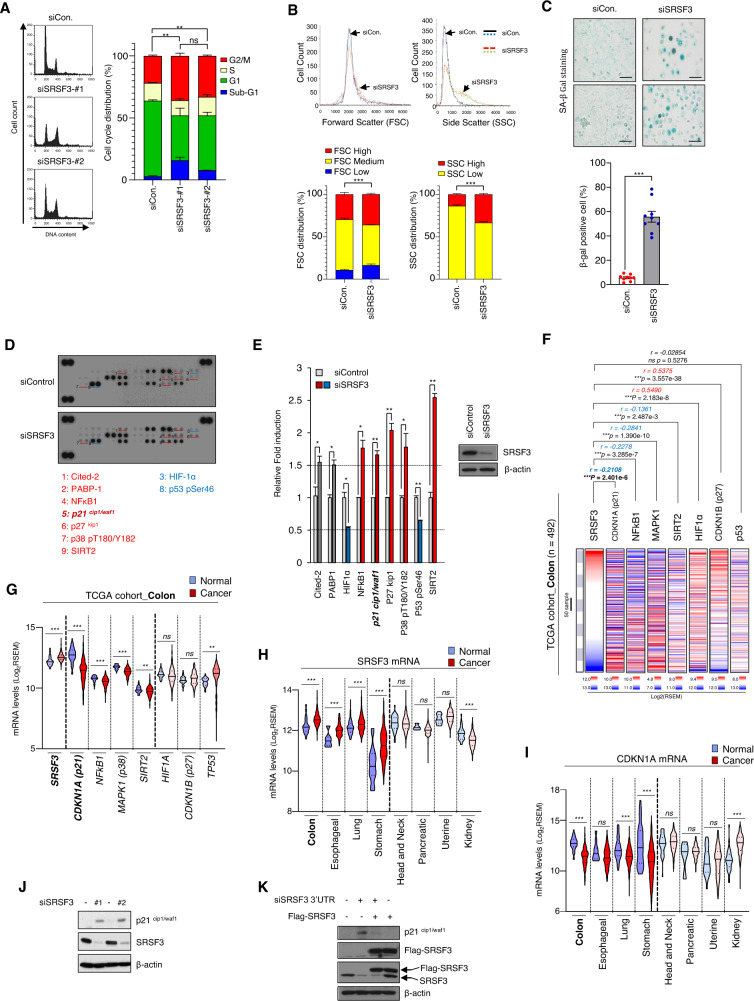
Silencing of SRSF3 caused p21^cip1/waf1^ upregulation and G2/M arrest in cellular senescence. **A** U2OS cells were transiently transfected with either control siRNA or two different SRSF3 siRNAs and then subjected to cell cycle analysis by flow cytometry. **B** Flow cytometer histograms forward scatter (FSC) and side scatter (SSC) for siControl or siSRSF3 transfection in SW480 cells. **C** Representative picture of SA-β-Gal staining in SW480 cells. Senescence β-Galactosidase staining kit was used for the assay according to the manufacturer’s protocol. Scale bar, 100 μm. **D** Whole cell lysates from siControl or SRSF3-depleted SW480 cells were incubated with human cell stress array membranes. Each pair of the most positive protein dots is numbered with the identification of the corresponding protein listed. **E** Levels of indicated protein were quantified after normalized control. Error bars indicate mean ± SD of spots. **F** Associations between SRSF3, CDKN1A, NFkB1, p38-MAPK1, SIRT2, HIF1a, CDKN1B, and TP53 levels and tumor tissues were identified by analyzing the TCGA-Colon datasets. Positive correlations are expressed as mean Spearman *r*, and *p* values are for a two-tailed Student’s *t*-test. **G** Expression of SRSF3, CDKN1A, NFkB1, p38-MAPK1, SIRT2, HIF1a, CDKN1B, and TP53 mRNA in colon cancer from TCGA database. Data are shown as mean ± SD. ns not significant; ***P < 0.001, two-tailed Student’s t-test. **H** Expression of SRSF3 mRNA in 8 primary cancer types from TCGA database. Data are shown as mean ± SD. ns, not significant; ***P < 0.001, two-tailed Student’s t-test. **I** Expression of p21^cip1/waf1^ (CDKN1A) mRNA in 8 primary cancer types from TCGA database. Data are shown as mean ± SD. ns not significant; ***P < 0.001, two-tailed Student’s t-test. **J** SW480 cells were transiently transfected with either control siRNA or two different SRSF3 siRNAs, and the levels of endogenous p21^cip1/waf1^, SRSF2, and β-actin were analyzed by western blotting. **K** The siSRSF3-3′UTR targeting 3′UTR region of SRSF3 mRNA was co-transfected with Flag-SRSF3 plasmid DNA construct in SW480 cells. Western blot analysis with anti- p21^cip1/waf1^, anti-Flag, anti-SRSF3, and anti-β-actin antibodies.

Previous studies have shown that SRSF3 deficiency inhibits cancer cell proliferation, migration, invasion, and metastasis. [9, 10, 36]. Consistently, knockdown of SRSF3 caused a decrease in BrdU staining, suggestive of decreased cellular proliferation (Fig. S1A). Through flow cytometric analysis, we found that knockdown of SRSF3 increased the cell population in the G2/M phase in SW480, a colon cancer cell line, and U2OS, a bone cancer cell line. (Figs. 1A and S1B). SRSF3 knockdown also increased the staining of Histone-H3 (Ser10), consistent with the arrest at the G2/M phase (Fig. S1C). An increase in the multi-nuclear formation during cell senescence can be a result of cell cycle termination during the G2/M arrest [30, 37, 38]. Moreover, deficiency of SRSF3 increased multi-nuclear formation (Fig. S1D).

Sustained arrest in the G2/M cell cycle phase is associated with cellular senescence [29, 30], which is accompanied by cell complexity and morphological changes [25, 26]. The cell complexity and size were analyzed using flow cytometry after SRSF3 knockdown in SW480 cells. In SRSF3 deficiency, there is a change in the cell size (FSC: forward scatter) which is small or large, and the cell complexity (SSC: side scatter) is significantly increased (Fig. 1B). Using SA-β-gal staining, we confirmed that SRSF3 deficiency promoted senescence in SW480 cells (Fig. 1C). As cellular senescence is accompanied by many changes in metabolic activity and the proteome [25, 26], proteomic changes caused by SRSF3 depletion were investigated using a cell stress array (R&D systems #ARY018). Compared to controls, cited-2, PABP-1, NFkB1, p21^cip1/waf1^, p27^kip1^, p38-MAPK1, and SIRT2 were upregulated while HIF1α and p53 were downregulated in SRSF3 knockdown cells (Figs. 1D, E and S2A). Moreover, we analyzed the correlation between SRSF3 and proteins with quantitative changes (1.5-fold increase or 0.5-fold decrease) upon SRSF3 knockdown in the TCGA-colon database; p21 (*r* = −0.2108, *p* = 2.401e−6), NFkB1 (*r* = −0.2278, *p* = 3.285e−7), p38 (*r* = −0.2841, *p* = 1.390e−10), and Sirt2 (*r* = −0.1361, *p* = 2.487e−3) show a statistically significant negative correlation with SRSF3. Conversely, p27 (*r* = 0.5375, *p* = 3.557e−38) was positively correlated, while p53 (*r* = −0.02854, *p* = 0.5276) was not statistically significant (Fig. 1F). Next, the expression between normal and cancer tissues was analyzed using the TCGA-colon database. In cancer tissues, compared to normal tissues, SRSF3 and p53 are upregulated, whereas p21, NFkB1, p38, and Sirt2 are downregulated. The HIF1α and p27 levels were not statistically significant (Fig. 1G). We identified four proteins (p21, NFkB1, MAPK1, and Sirt2) as potential target gene candidates regulated by SRSF3 through cell stress array analysis, correlation analysis, and expression levels between cancer and normal tissues. Given that SRSF3 deficiency leads to cellular senescence and G2/M arrest, we supposed that p21 may be a direct target of SRSF3. Thus, we further analyzed any potential correlation of mRNA levels between SRSF3 and p21 in eight cancers using TCGA database. In colon, esophagus, lung, and stomach cancer tissues, the SRSF3 mRNA level was upregulated compared to that in the normal tissues (Fig. 1H). Conversely, the p21 mRNA level in the colon, esophagus, lung, and stomach was downregulated in cancer tissues compared to that in normal tissues (Fig. 1I). In the head and neck, pancreas, and uterus, where SRSF3 did not differ between normal and cancerous tissues, the p21 mRNA level was also not statistically significant (Fig. 1H, I). To affirm that the change in mRNA levels correlate with protein levels, we analyzed the results published by Fredrik Pontén et al. [39] where the protein levels of SRSF3 and p21 were compared using tissue microarray and cell microarray. Similar to the TCGA mRNA analysis, SRSF3 was highly expressed, whereas p21 was low expressed (Fig. S2C and Tables S1, 2). These results altogether indicate that the expressions of SRSF3 and p21 are inversely correlated in various cancers. When we knockdown SRSF3 with two independent siRNAs, p21 increases were observed, suggesting a causal relationship between SRSF3 and p21 (Figs. 1J and S2C). The increase in p21 was reversed by re-expressing siRNA-resistant SRSF3, ruling out any off-target effects (Fig. 1K).

### Upregulation of p21 caused by SRSF3 deficiency is p53-independent

**Fig. 2:**
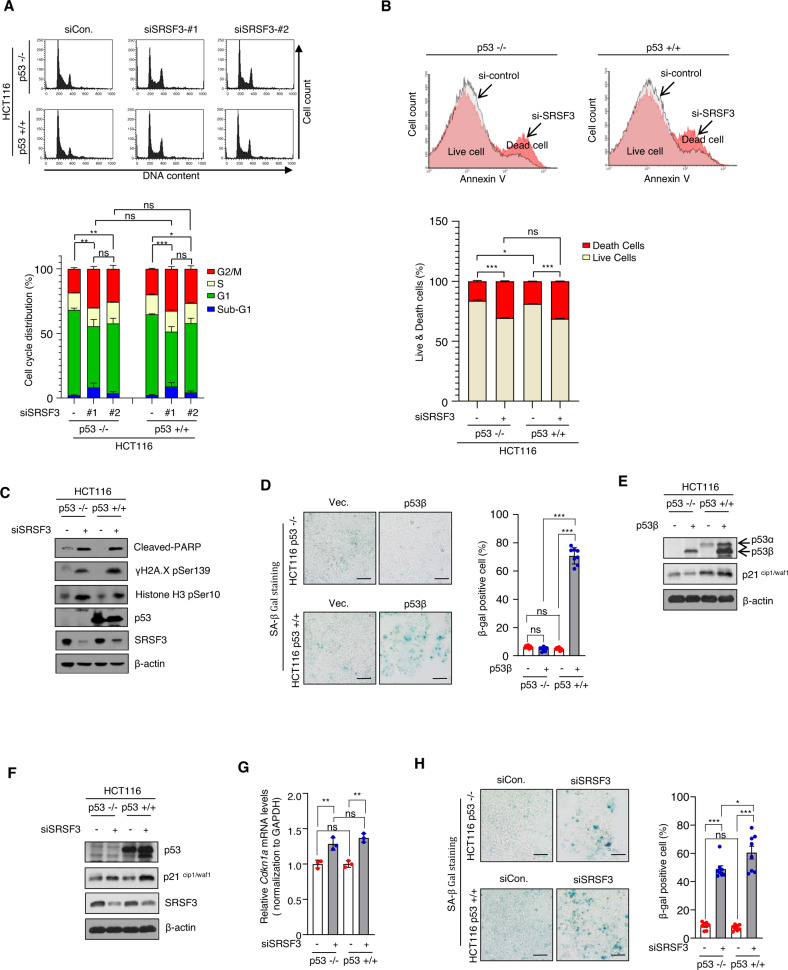
Depletion of SRSF3 induced apoptosis, senescence, and an increase in p21 ^cip1/waf1^ was independent of p53. **A** HCT116 p53−/− and p53+/+ cells were transfected with siControl or siSRSF3 #1, #2 and subjected to cell cycle analysis by flow cytometry. **B** Silencing of SRSF3 in HCT116 p53−/− or p53+/+ cells, which were stained with annexin V and PI, and subjected to flow cytometry. **C** The siControl or siSRSF3 were transfected into HCT116 p53−/− or p53+/+ cells for 72 h and analyzed by Western blot analysis for the indicated antibodies. **D** After transfection of p53−/− or p53+/+ HCT116 cells with empty vector or p53β plasmid DNA for 36 h, cells were stained with SA-β Galactosidase. Scale bar, 100 μm. **E** Cell lysates were prepared from the HCT116 p53−/− or p53+/+ cells after transfection with p53β plasmid DNA for 36 h. The level of p53 α, β, p21^cip1/waf1^, and β-actin were measured by western blot analysis. **F** The siControl or siSRSF3 were transfected into HCT116 p53−/− or p53+/+ cells for 72 h and analyzed by Western blot analysis for the indicated antibodies. **G** The levels of p21 mRNA in HCT116 p53−/− or p53+/+ cells transfected with either control or SRSF3 siRNA. Data are shown as mean ± SD. ***P < 0.001, two-tailed Student’s *t*-test. **H** SA-β-Gal staining in HCT116 p53−/− or p53+/+ cells after 72 h of siControl or siSRSF3 treatment, Scale bar, 100 μm.

The gene, p21 is one of the most representative target genes of p53 [31]. However, SRSF3 depletion had opposite effects on p21 and p53 (see Fig. 1D; p21 is upregulated, while p53 pSer46 is downregulated). Furthermore, there is no change of SRSF3 expression levels in p53 activation or overexpression conditions where p21 levels are upregulated (Fig. S3A–D). These results suggest that the effect of SRSF3 on p21 occurs independently of p53. To test this, we depleted SRSF3 using two independent SRSF3 siRNAs in p53 WT (HCT116 p53+/+) and p53 null cells (HCT116 p53−/−). In HCT116 p53+/+ cells, SRSF3 knockdown leads to an increase in the number of cells in the Sub-G1 and G2/M phases. Similarly, the sub-G1 and G2/M phases increased in SRSF3-depleted HCT116 p53−/− cells (Fig. 2A). Therefore, SRSF3 knockdown resulted in increased sub-G1 and G2/M cell cycle arrest in a p53-independent manner. Moreover, persistent G2/M cell cycle arrest induces apoptosis [40]. Therefore, we analyzed the apoptosis of HCT116 p53+/+ and HCT116 p53−/− cells using flow cytometry analysis by performing Annexin V staining after knockdown of SRSF3 for 72 h. Similar to Fig. 2a, SRSF3 knockdown induced apoptosis independently of p53 (Fig. 2B). Moreover, SRSF3 deficiency in HCT116 cells increased the levels of cleaved-PARP, γH2A.X (Ser139), and Histone-H3 (ser10) independent of p53 (Fig. 2C). The generation of p53β according to the alternative splicing of p53α is known to induce cellular senescence, and SRSF3 is involved in the generation of p53β in the nucleus [41]. We tested whether the induction of cellular senescence by p53β overexpression is p53α-dependent. To confirm this, we overexpressed p53β in HCT116 p53+/+ and HCT116 p53−/− cells and analyzed cellular senescence using SA-β-gal staining. The induction of senescence by p53β is p53α-dependent (Fig. 2D). Next, we tested whether the overexpression of p53β induced an increase in p21 expression. It was found that overexpression of p53β in HCT116 p53+/+ and HCT116 p53−/− cells did not induce an increase in p21 expression (Fig. 2E). We also tested whether an increase in p21 caused by SRSF3 depletion was p53-dependent. To prove this, we knocked down SRSF3 in HCT116 p53+/+ and HCT116 p53−/− cells. In p53 WT and p53 null cells, p21 protein increased due to SRSF3 deficiency, and p21 mRNA marginally increased (Fig. 2F, G). Correspondingly, in HCT116 p53+/+ and HCT116 p53−/− cells, senescence induction due to SRSF3 depletion was p53-independent, as assessed by SA-β-gal staining (Fig. 2H). These results altogether suggest that induction of p21 and cellular senescence caused by SRSF3 depletion are independent of p53 status.

### SRSF3 inhibits the translation of p21 mRNA

**Fig. 3:**
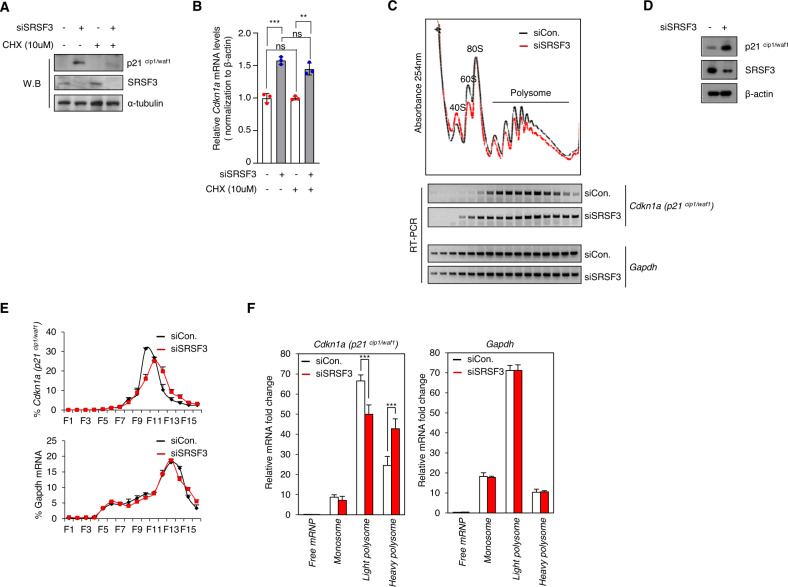
SRSF3 regulates translation of p21^cip1/waf1^ mRNA in cytoplasm. **A**, **B** After treatment with siControl or siSRSF3 for 72 h, SW480 cells were untreated or treated with 10 μM cycloheximide (CHX) for 24 h. Data are shown as mean ± SD. ns, not significant; ***P < 0.001, **p < 0.01, two-tailed Student’s t-test. **C** Cells were transfected with control and SRSF3 siRNAs and separated by polysome fraction. RNA samples from each fraction were subjected to RT-PCR analysis to detect CDKN1A (p21^cip1/waf1^). GAPDH was used as control. **D** Western blot analysis was performed with whole cell extract from (**C**). Quantification of RT-PCR analysis from (**C**) was performed to observe relative abundance for Cdkn1a and GAPDH in each fraction (**E**). Free mRNP (Fraction 1–3), monosome (Fraction 4–8), light polysome (Fraction 9–11), and heavy polysome (Fraction 12–16) (**F**). Data are shown as mean ± SD. ***P < 0.001, two-tailed Student’s t-test.

Intriguingly, SRSF3 deficiency only caused a marginal increase in p21 mRNA (Fig. S4). Moreover, as p21 regulation is p53-independent, whether an increase in p21 is caused by SRSF3 deficiency at the translational level, as observed with respect to PDCD4, needs to be investigated [10]. Moreover, an increase in p21 protein level upon SRSF3 knockdown was no longer observed when the cells were treated with cycloheximide (CHX), an inhibitor of translation (Fig. 3A). CHX treatment did not change the mRNA level of p21 (Fig. 3B). One way to interpret this is that SRSF3-mediated control of p21 levels occurs, in part, through translation. Therefore, we hypothesized that the translation efficiency of p21 mRNA is altered by SRSF3 depletion. To test this hypothesis, we employed the polysome profiling analysis to determine any changes in the translation efficiency of p21 mRNA with or without SRSF3 knockdown in SW480 cells. In SRSF3-depleted cells, p21 mRNA moved from light to heavy polysome fractions compared to the control cells. The shift to heavy polysomes suggests an increase in translation efficiency. This change was not observed for GAPDH mRNA (Fig. 3C–F), suggesting that this translational control is selective for specific mRNAs, including p21. These results suggest that SRSF3 negatively regulates p21 mRNA translation.

### SRSF3 interacts and colocalizes with eIF4A1 in the cytoplasm

**Fig. 4:**
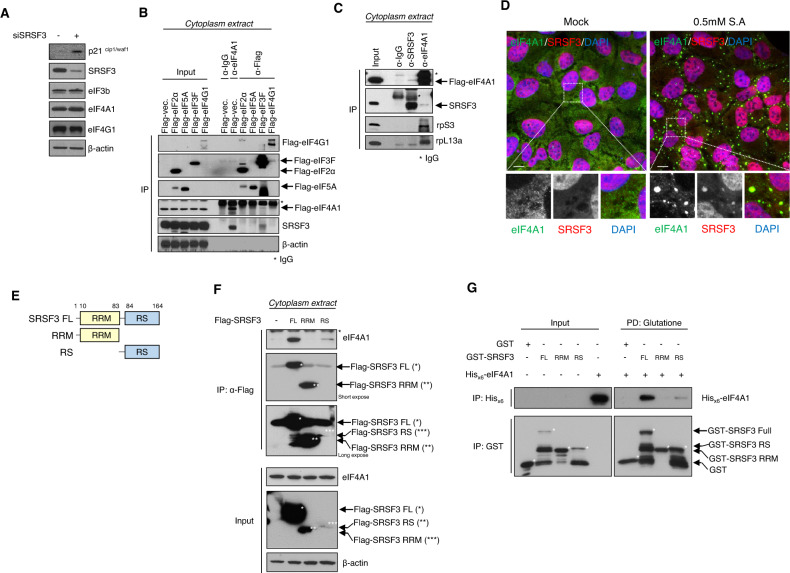
SRSF3 interacts with eIF4A1 in the cytoplasm. **A** The siControl or siSRSF3 treated SW480 cells were detected SRSF3, p21^cip1/waf1^ and translation initiation factor proteins. The protein, β-actin was used as a loading control. **B** HEK-293T cells transfected with Flag-eIF2α, Flag-eIF5A, Flag-eIF3F, and Flag-eIF4G1. Co-immunoprecipitation was carried out using the anti-Flag antibody or eIF4A1 antibody. Levels of indicated proteins in cytoplasmic extract and Co-IP products were analyzed by western blot analysis. **C** Cytoplasmic extract of SW480 cells were immunoprecipitation with an anti-SRSF3 or anti-eIF4A1 antibody. The immunocomplexes and input were analyzed using western blot with anti-Flag, anti-SRSF3, anti-rpS3, and anti-rpL13a antibodies. **D** U2OS cells were treatment with or without sodium arsenite (0.5 mM, 1 h) for the mentioned time period before for immunofluorescence microscopy. Cells were stained with eIF4A1 (Green), SRSF3 (Red). Scale bar, 10 μm. **E** Schematic representation of full length and a series of deletion mutants of SRSF3. **F** Lysates from HEK293T cells transfected with Flag-tagged full length (FL) or deletion mutant SRSF3 were immunoprecipitated with anti-Flag antibody and subjected to western blot analysis with anti-Flag, anti-eIF4A, and anti-β-actin antibodies. **G** In vitro GST-pulldown assay of the binding of recombinant His_x6_-tagged eIF4A1 with GST or the GST-SRSF3 (FL, RRM, and RS domain).

The key rate-limiting step in the translation process is initiation by initiating factors [42, 43]. SRSF3 may control the translation initiation step by affecting the levels of translation-initiating factors. This may not be the case, as depletion of SRSF3 does not alter the expression levels of eIF4G, eIF4A, or eIF3b, which are known as major translation initiation factors (Fig. 4A). We also tested whether SRSF3 is associated with translation initiation factors. When we separated the nuclear and cytoplasmic fractions, a portion of SRSF3 is found in cytoplasm, supporting a possible role of SRSF3 in translation control (Fig. S5A–C). FLAG-tagged eIF2α, eIF5A, eIF3F, and eIF4G1 proteins were transiently expressed in HEK293T cells, and cytoplasmic extracts were isolated before co-immunoprecipitation with anti-eIF4A1 or anti-FLAG antibodies. We found that SRSF3 was strongly associated with eIF4A1 (Fig. 4B, C). Sodium arsenite treatment enables translation initiation factors to migrate and form aggregates such as stress granules (SG) and processing bodies (PB) [44]. Indeed, we found that sodium arsenite treatment caused localization of eIF4A1 and SRSF3 into these cytoplasmic RNA granules (Fig. 4D). This suggests that these two proteins interact physically to exert translational control in the cytoplasm and co-aggregate into translationally stalled those RNA granules upon stress.

### The RS domain of SRSF3 is responsible for interacting with eIF4A1

SRSF3 contains an RNA recognition motif (RRM) that plays a role in RNA binding at the N-terminus and an arginine/serine-rich (RS) domain that interacts with other proteins at the C-terminus [45, 46]. As analyzing protein-binding sites can provide clues to the nature of protein interactions [47], we tested which domain of SRSF3 binds to eIF4A1 using SRSF3 WT and deletion constructs (Fig. 4E). FLAG-tagged SRSF3_full-length (FL), RRM, and RS domain mutants were transfected into HEK293T cells, followed by immunoprecipitation with an anti-FLAG antibody. The FLAG-tagged SRSF3_FL immunoprecipitated eIF4A1, while SRSF3_RRM did not (Fig. 4F). The SRSF3-RS could immunoprecipitate eIF4A1 efficiently, even though its expression is very low compared to FL and RRM (see lane 4 of input). To test if SRSF3 directly binds eIF4A1, we performed a GST pull-down analysis using the recombinant GST-SRSF3 proteins and the His_x6_-eIF4A1 protein. Similar to the results of immunoprecipitation, GST-SRSF3_FL and RS domains directly bound His_x6_-eIF4A1. However, no binding was detectable between the GST-SRSF3_RRM domain and His_x6_-eIF4A1 (Fig. 4G). These results suggest that SRSF3 interacts with eIF4A1 and that the RS domain of SRSF3 plays an important role in interacting with translation factors such as eIF4A1.

### SRSF3 inhibits the function of eIF4A1 at the 3′UTR region of p21 mRNA

**Fig. 5:**
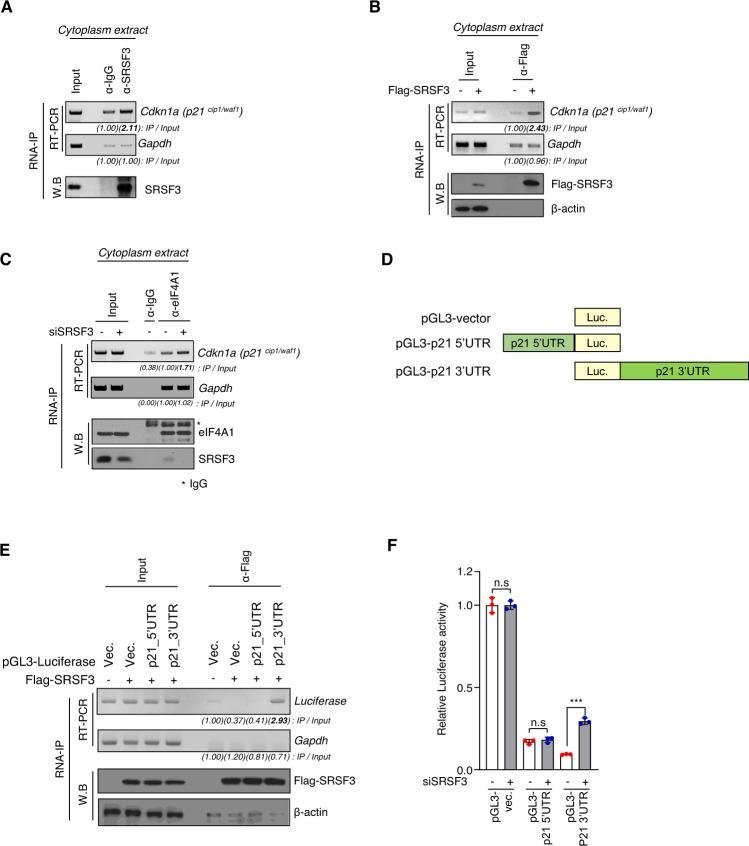
SRSF3 represses p21 translation though interacts p21^cip1/waf1^ 3′UTR region. **A** RNP-immunoprecipitation (RNP-IP) analysis was performed using a IgG (mouse) or an anti-SRSF3 antibody on cytoplasm extract from SW480 cells, followed by RT-PCR and western blot analysis. **B** HEK293T cells transfected with Flag-SRSF3. RNP-IP analysis was carried out by using the anti-Flag antibody, followed by RT-PCR and western blot analysis. **C** SW480 cells treated with siControl or siSRSF3 siRNA. RNP-IP analysis were carried out using an IgG or an anti-eIF4A1 antibody. It was followed by western blot and RT-PCR analysis. **D** A schematic of firefly luciferase constructs in which the 5′UTR and 3′UTR of p21^cip1/waf1^ were cloned upstream or downstream of the luciferase coding region (Luc.). **E** HEK293T cells were transfected with pGL3-vector, pGL3-p21cip1/waf1 5′UTR, and pGL3-p21cip1/waf1 3′UTR plasmids and subjected to RNP-immunoprecipitation analysis as described for panel. **F** Luciferase assays were performed after transfecting pGL3-vector, pGL3-p21 5′UTR and pGL3-p21 3′UTR construct into SW480 cells transfected with either siControl or siSRSF3. Renilla luciferase vector was co-transfected for normalization.

To determine the mechanistic interplay between SRSF3 and eIF4A in p21 mRNA translation, we tested whether SRSF3 binds directly to p21 mRNA in the cytoplasm. The cytoplasmic extract of SW480 was used to perform RNP-immunoprecipitation (RNP-IP) using an anti-SRSF3 antibody. While there was no enrichment of GAPDH mRNA in the SRSF3 immunoprecipitates compared to the control IP, there was a clear enrichment of p21 mRNA in the SRSF3 IP (Fig. 5A). Similarly, FLAG-SRSF3 immunoprecipitated with p21 mRNA, but not with the GAPDH mRNA (Fig. 5B). Next, we tested whether SRSF3 affected the binding ability of eIF4A1 to p21 mRNA using RNA-IP experiments. The ability of eIF4A to bind to p21 mRNA enhanced upon SRSF3 knockdown (Fig. 5C). Moreover, SW480 cells were subjected to serum starvation for 72 h to induce p21 expression, and cell extracts were immunoprecipitated with an anti-SRSF3 antibody. As expected, SRSF3 and eIF4A1 did not bind under serum-starvation conditions (Fig. S6). In the translation control process, RNA-binding proteins typically bind to the 5′UTR or 3′UTR of p21 mRNA and regulate translation through mutual regulation with translation regulators [48–50]. To determine the binding region of SRSF3 on the p21 mRNA, we generated constructs in which either the 5′UTR or 3′UTR region of the p21 mRNA was fused with luciferase (Fig. 5D). Using RNA-IP experiments, we found that SRSF3 specifically bound to the 3′UTR region, but not to the 5′UTR region (Fig. 5E). Moreover, knockdown of SRSF3 enhanced luciferase expression only when it was fused to the 3′UTR of p21 mRNA, but not to the 5′UTR (Fig. 5F). Thus, these results suggest that SRSF3 inhibits p21 expression by directly binding to the 3′UTR region of p21 mRNA, wherein it antagonizes eIF4A1 recruitment.

## Discussion

In the present study, we uncovered a new regulatory mode of p21 expression that occurs via SRSF3-mediated translation control. We found that: (1) SRSF3 and p21 expression levels are inversely correlated using the TCGA dataset, (2) SRSF3 depletion causes upregulation of p21 expression, cell cycle arrest, and cellular senescence, (3) the induction of p21 is independent of p53, and (4) SRSF3 directly regulates the translation of p21 mRNA through the 3′UTR.

The novel role of the SR family proteins in translation regulation began approximately fifteen years ago. These are nucleocytoplasmic shuttling SR proteins, such as SRSF1, SRSF3, and SRSF7 [10, 20, 21]. The initial finding that SRSF3 may potentially function in cytoplasmic RNA metabolism was obtained from the siRNA screen to identify genes that are involved in cytoplasmic RNA granule formation [51]. RNA granules are mRNP aggregates in which translationally silenced mRNAs are deposited. SRSF3 depletion potently ablates RNA granules under normal and stress conditions and is an RNA granule component [52]. Moreover, SRSF3 was found to be associated with translating ribosomal fractions similar to SRSF1 and SRSF7, indicating its possible role in mRNA translation [52]. Direct evidence of SRSF3-mediated translation control was obtained from our previous study, where SRSF3 inhibited PDCD4 mRNA translation by directly binding to the 5′UTR region [10]. In this study, unlike PDCD4, p21 mRNA translation was found to be repressed by SRSF3 through 3′UTR binding. Moreover, this inhibitory mode seems to occur through interaction with eIF4A, a translation initiation factor. Observing that this interaction is abolished under serum starvation conditions where p21 expression is upregulated, we propose that SRSF3-mediated repression of p21 mRNA translation is likely through inhibition of eIF4A function. One possibility is that under serum starvation stress, SRSF3 could be subjected to post-translational modification (e.g., NEDDylation), by which its interaction with eIF4A is abolished, thereby reinitiating the silenced p21 mRNA translation [53].

The C-terminal RS domain of SRSF3 promotes protein–protein interactions to facilitate its function [45, 46]. SRSF3 has two isoforms: Isoform 1 produces a full-length protein and isoform 2 produces SRSF-TR, in which two-thirds of the RS domain is truncated. Previously, depletion of SLU7, PTBP1, and PTBP2 or overexpression of RBM4 has been reported to induce SRSF3-TR [54–57], which causes upregulation of p21 [55, 58]. Our study shows that SRSF3-TR mediated upregulation of p21 expression is due to the lack of an RS domain by which eIF4A function could be inhibited (Fig. 4).

The p53-independent regulation of p21 expression by SRSF3 is rather surprising because the p53-mediated regulation of p21 is well known [31]. Moreover, a recent report has demonstrated that SRSF3 depletion induces p53β, an alternatively spliced isoform of p53, and promotes cellular senescence. Hence, whether SRSF3 depletion-mediated p21 upregulation and cellular senescence are due to p53 (including p53β) was investigated using a p53 knockout cell line. We found that: (1) SRSF3 knockdown causes DNA damage response, G2/M arrest, and cellular senescence in HCT116 p53−/− cells as in WT; (2) p53β itself does not induce cellular senescence in HCT116 p53−/−; and (3) the induction of p21 upon SRSF3 depletion also occurred in HCT116 p53−/− cells, although the level was somewhat less than that in the WT. These results suggest that the induction of p21 and cellular senescence caused by SRSF3 depletion are independent of the p53 pathway.

**Fig. 6:**
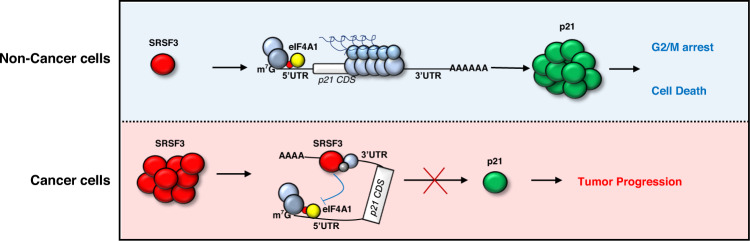
A model diagram of SRSF3-mediated post-transcriptional regulation of p21 mRNA. In cancer cells, upregulation of SRSF3 expression inhibits the translation of p21 mRNA through directly binding at 3′UTR, leading to tumor progression. In non-cancerous cells, low level of SRSF3 relieves the translation of p21 mRNA in normal or certain stress conditions, leading to cell cycle arrest and death.

Thus, our study identified a new mode of p21 expression control. Our work also adds to the growing list of splicing-independent roles of SR proteins in the cytoplasm. Our results also imply that inhibiting the interaction between SRSF3 and eIF4A1 is a possible therapeutic approach for inducing p21-dependent cellular senescence in cancer cells (Fig. 6).

## Supplementary information

Supplementary Fig. 1

Supplementary Fig. 2

Supplementary Fig. 3

Supplementary Fig. 4

Supplementary Fig. 5

Supplementary Fig. 6

Supplementary Table 1

Supplementary Table 2

Supplementary Table 3,4 and 5

Original data file_1

Original data file_2

Original data file_3

Original data file_4

Original data file_5

Reproducibility checklist

## Data Availability

All datasets generated and analyzed during this study are included in this published article and its Supplementary information files. Additional data are available from the corresponding author on reasonable request.
