# Supplementary material for: Splicing factor SRSF3 represses translation of p21cip1/waf1 mRNA
Source: Cell Death Dis. 2022 Nov 7;13(11):933. doi: 10.1038/s41419-022-05371-x (PMC9640673; doi:10.1038/s41419-022-05371-x)
Supplement: Supplementary file 1 — Supplementary Fig. 1 [file 41419_2022_5371_MOESM1_ESM.pdf]

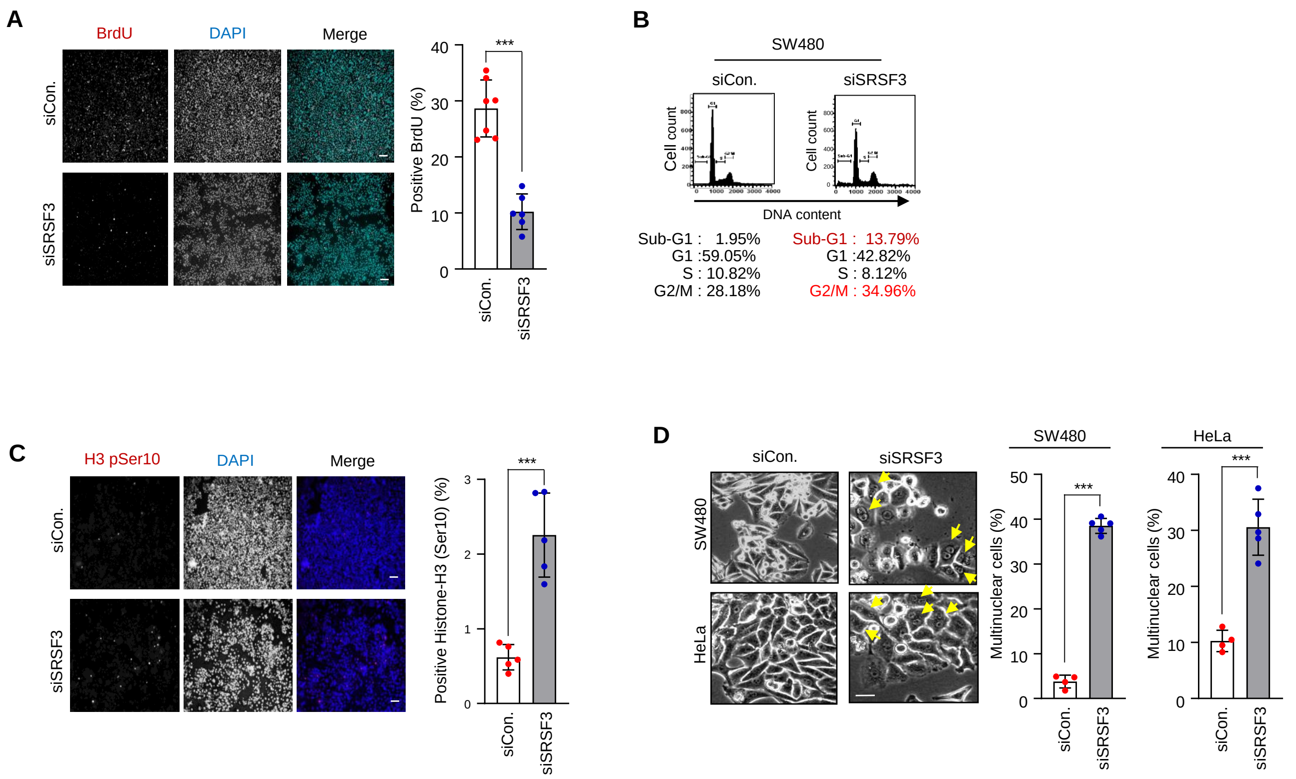

Supplementary Fig. 1 Deficiency of SRSF3 causes a variety of cellular changes. A. Immunofluorescent staining with anti-BrdU antibody of siControl or siSRSF3 SW480 cells. Nuclei were stained with DAPI (blue). Scale bars = 100 μm. **B.** SW480 cells were transfected with siControl or siSRSF3 and subjected to cell cycle analysis by flow cytometry. **C.** Immunofluorescent staining with the anti- Histone-H3 (Ser10) antibody (red) of siControl or siSRSF3 SW480 cells. Nuclei were stained with DAPI (blue). Scale bars = 100 μm. **D.** Silencing of SRSF3 cells were subjected to microscopy and multinuclear cell count. Scale bar = 40μm. Data are shown as mean ± SD. ns, not significant; ***P < 0.001, two-tailed Student’s t-test.
