# Supplementary material for: Splicing factor SRSF3 represses translation of p21cip1/waf1 mRNA
Source: Cell Death Dis. 2022 Nov 7;13(11):933. doi: 10.1038/s41419-022-05371-x (PMC9640673; doi:10.1038/s41419-022-05371-x)
Supplement: Supplementary file 2 — Supplementary Fig. 2 [file 41419_2022_5371_MOESM2_ESM.pdf]

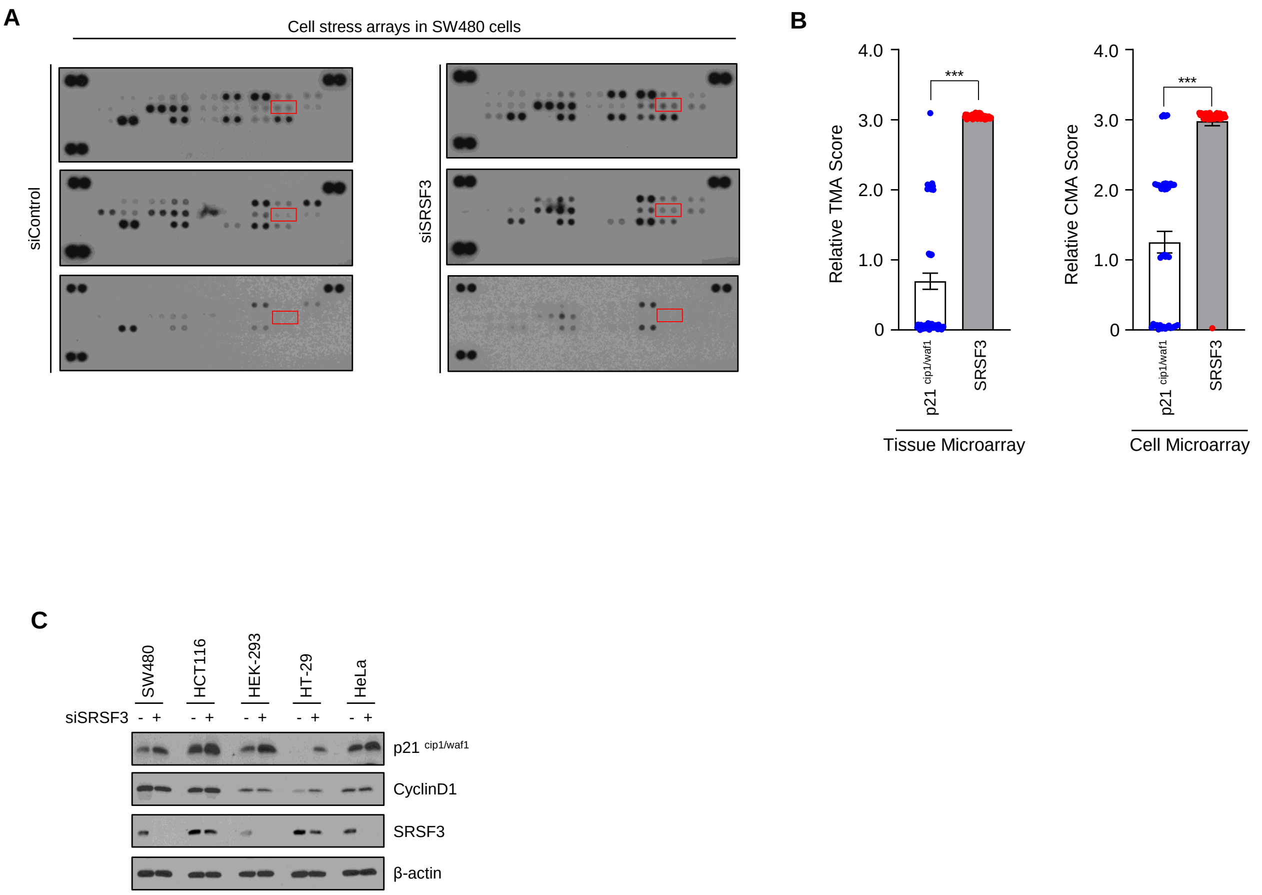

Supplementary Fig. 2 SRSF3 is associated with the expression of p21. A. Whole cell lysates from siControl or SRSF3-depleted SW480 cells were incubated with human cell stress array membranes. **B.** Comparison of p21 and SRSF3 protein levels in tissue microarray and cell microarray. **C.** Cell lysates were prepared from the SW480, HCT116, HEK293, HT-29, and HeLa cells after transfected with siControl or siSRSF3 for 72 h.
