# Supplementary material for: Splicing factor SRSF3 represses translation of p21cip1/waf1 mRNA
Source: Cell Death Dis. 2022 Nov 7;13(11):933. doi: 10.1038/s41419-022-05371-x (PMC9640673; doi:10.1038/s41419-022-05371-x)
Supplement: Supplementary file 3 — Supplementary Fig. 3 [file 41419_2022_5371_MOESM3_ESM.pdf]

Sup Fig.3

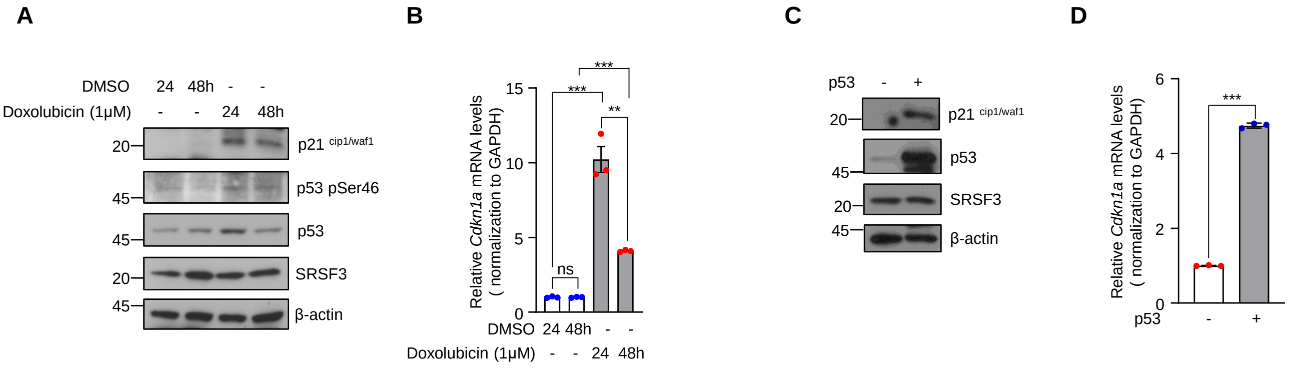

Supplementary Fig. 3 The increase in p21 by p53 was not associated with SRSF3. A. HEK293T cells were treated with 1μM doxorubicin for different times. Analyzed by Western blot analysis for the indicated antibodies. **B.** The levels of p21 mRNA in HEK293T cells treated with 1uM doxorubicin for different times. Data are shown as mean ± SD. ***$P < 0.001$, **$P < 0.005$, two-tailed Student's *t*-test. **C.**HEK293T cells were transfected with p53 plasmid DNA. The p21, p53, SRSF3 and β-actin were analyzed by western blotting using the indicated antibodies. **D.** The levels of p21 mRNA in HEK293T cells transfected with p53 plasmid DNA. Data are shown as mean ± SD. ***$P < 0.001$, two-tailed Student's *t*-test.
