# Supplementary material for: Splicing factor SRSF3 represses translation of p21cip1/waf1 mRNA
Source: Cell Death Dis. 2022 Nov 7;13(11):933. doi: 10.1038/s41419-022-05371-x (PMC9640673; doi:10.1038/s41419-022-05371-x)
Supplement: Supplementary file 4 — Supplementary Fig. 4 [file 41419_2022_5371_MOESM4_ESM.pdf]

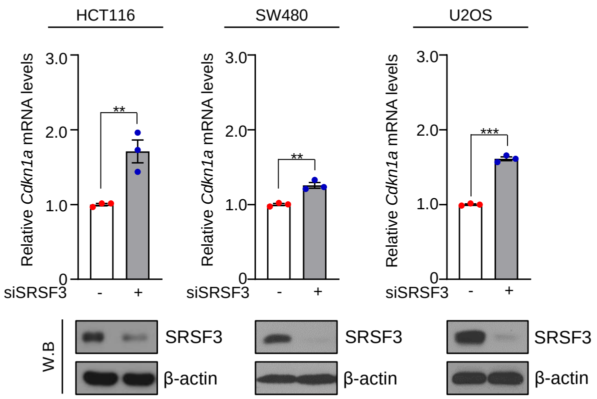

Supplementary Fig. 4 Deficiency of SRSF3 slightly increases p21 mRNA. The levels of p21 mRNA in HCT116, SW480, U2OS cells transfected with either control or SRSF3 siRNA. Data are shown as mean ± SD. ***$P < 0.001$, **$P < 0.005$ two-tailed Student's *t*-test.
