# Supplementary material for: Splicing factor SRSF3 represses translation of p21cip1/waf1 mRNA
Source: Cell Death Dis. 2022 Nov 7;13(11):933. doi: 10.1038/s41419-022-05371-x (PMC9640673; doi:10.1038/s41419-022-05371-x)
Supplement: Supplementary file 5 — Supplementary Fig. 5 [file 41419_2022_5371_MOESM5_ESM.pdf]

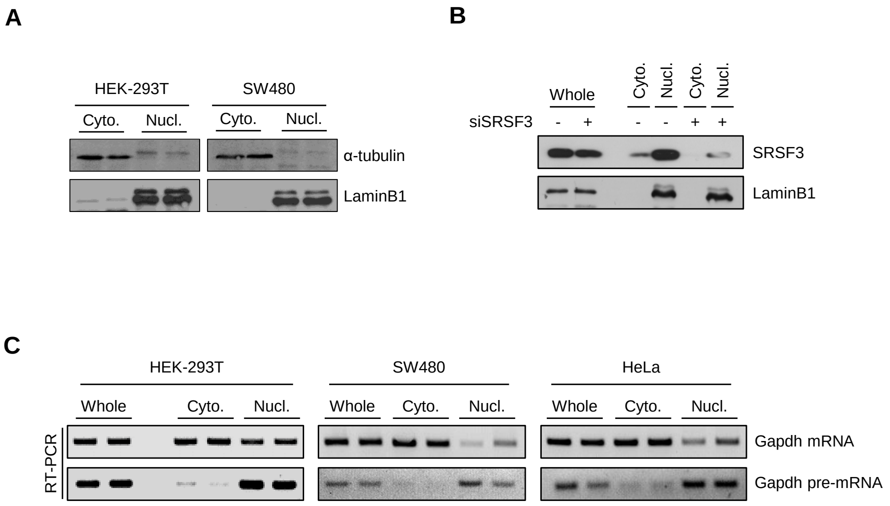

Supplementary Fig. 5 Separation of cytoplasmic and nuclear isolates. A. The cytoplasm and nucleus are clearly separated from HEK293T and SW480 cells. **B.** Separation of cytoplasm and nucleus from cells transfected with siControl or siSRSF3 for 72 h. C. Whole, cytoplasmic, and nuclear RNAs from HEK293T, SW480, and HeLa cells were prepared with Trizol and analyzed by RT-PCR. GAPDH and GAPDH pre-mRNA were used as cytoplasm and nuclear fraction controls, respectively.
