# Supplementary material for: Splicing factor SRSF3 represses translation of p21cip1/waf1 mRNA
Source: Cell Death Dis. 2022 Nov 7;13(11):933. doi: 10.1038/s41419-022-05371-x (PMC9640673; doi:10.1038/s41419-022-05371-x)
Supplement: Supplementary file 6 — Supplementary Fig. 6 [file 41419_2022_5371_MOESM6_ESM.pdf]

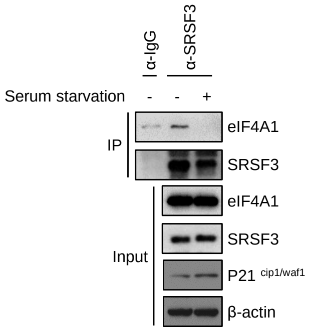

Supplementary Fig. 6 Serum starvation reduces the interaction between SRSF3 and eIF4A1. SW480 cells were serum starvated for 72 hours. Cytoplasm extract was obtained through immunoprecipitation with an anti-SRSF3 antibody. The immunocomplexes and inputs were analyzed by western blot with indicated antibodies.
