# Supplementary material for: Splicing factor SRSF3 represses translation of p21cip1/waf1 mRNA
Source: Cell Death Dis. 2022 Nov 7;13(11):933. doi: 10.1038/s41419-022-05371-x (PMC9640673; doi:10.1038/s41419-022-05371-x)
Supplement: Supplementary file 7 — Supplementary Table 1 [file 41419_2022_5371_MOESM7_ESM.pdf]

Supplementary Table 1 Tissue microarray of p21 and SRSF3

tissue	p21			SRSF3		
	intensity	fraction	summary_expression_value	intensity	fraction	summary_expression_value
Adrenal	strong	<25%	moderate	strong	>75%	strong
Appendix, glandular cells	negative		negative	strong	>75%	strong
Appendix, lymphocytes	negative		negative	strong	>75%	strong
Bladder, urothelial cells	moderate	75%-25%	moderate	strong	>75%	strong
Bone marrow	strong	rare	moderate	strong	>75%	strong
Brain cerebral cortex	negative		negative	strong	>75%	strong
Brain cerebral cortex, neurons	negative		negative	strong	>75%	strong
Brain hippocampus, glial	negative		negative	strong	>75%	strong
Brain hippocampus, neurons	negative		negative	strong	>75%	strong
Breast, glandular cells	strong	rare	moderate	strong	>75%	strong
Bronchus, glandular cells	strong	<25%	moderate	strong	>75%	strong
Cerebellum, granular layer	negative		negative	strong	>75%	strong
Cerebellum, molecular layer	negative		negative	strong	>75%	strong
Cerebellum, neurons	negative		negative	strong	>75%	strong
Cervix, glandular cells	moderate	<25%	weak	strong	>75%	strong
Cervix, squamous	moderate	75%-25%	moderate	strong	>75%	strong
Colon, glandular cells	moderate	75%-25%	moderate	strong	>75%	strong
Duodenum, glandular cells	weak	<25%	negative	strong	>75%	strong
Epididymis, glandular cells	strong	<25%	moderate	strong	>75%	strong
Esophagus, squamous	moderate	<25%	weak	strong	>75%	strong
Fallopian tube, glandular cells	negative		negative	strong	>75%	strong
Gallbladder, glandular cells	negative		negative	strong	>75%	strong
Heart muscle, myocytes	negative		negative	strong	>75%	strong
Kidney, glomeruli	negative		negative	strong	>75%	strong
Kidney, tubules	negative		negative	strong	>75%	strong
Left ventricle, glial	negative		negative	strong	>75%	strong
Left ventricle, neurons	negative		negative	strong	>75%	strong
Liver, glandular cells	negative		negative	strong	>75%	strong
Liver, hepatocytes	negative		negative	strong	>75%	strong
Lung, macrophages	weak	>75%	weak	strong	>75%	strong
Lung, pneumocytes	negative		negative	strong	>75%	strong
Lymph node, germinal center	negative		negative	strong	>75%	strong
Lymph node, other lymphoid	negative		negative	strong	>75%	strong
Nasopharynx, glandular cells	moderate	<25%	weak	strong	>75%	strong
Oral mucosa, squamous	moderate	75%-25%	moderate	strong	>75%	strong
Ovary, stromal cells	negative		negative	strong	>75%	strong
Pancreas, glandular cells	negative		negative	strong	>75%	strong
Pancreas, islet cells	negative		negative	strong	>75%	strong
Parathyroid gland, glandular	weak	rare	negative	strong	>75%	strong
Placenta, decidual cells	negative		negative	strong	>75%	strong
Placenta, trophoblasts	moderate	>75%	moderate	strong	>75%	strong
Prostate, glandular cells	negative		negative	strong	>75%	strong
Rectum, glandular cells	moderate	75%-25%	moderate	strong	>75%	strong
Salivary gland, glandular cells	negative		negative	strong	>75%	strong
Seminal vesicle, glandular	strong	rare	moderate	strong	>75%	strong
Skin I, epidermal cells	moderate	75%-25%	moderate	strong	>75%	strong
Skin II, epidermal cells	strong	75%-25%	strong	strong	>75%	strong
Small intestine, glandular cells	moderate	75%-25%	moderate	strong	>75%	strong
Smooth muscle	negative		negative	strong	>75%	strong
Spleen, red pulp	negative		negative	strong	>75%	strong
Spleen, white pulp	negative		negative	strong	>75%	strong
Stomach I, glandular cells	negative		negative	strong	>75%	strong
Stomach II, glandular cells	negative		negative	strong	>75%	strong
Striated muscle	negative		negative	strong	>75%	strong
Testis, leydig cells	negative		negative	strong	>75%	strong
Testis, seminiferus duct	strong	rare	moderate	strong	>75%	strong
Thyroid gland, glandular cells	moderate	<25%	weak	strong	>75%	strong
Tonsil, germinal center	negative		negative	strong	>75%	strong
Tonsil, other lymphoid cells	negative		negative	strong	>75%	strong
Tonsil, squamous	moderate	75%-25%	moderate	strong	>75%	strong
Uterus I, endometrial stroma	negative		negative	strong	>75%	strong
Uterus I, glandular cells	negative		negative	strong	>75%	strong
Uterus II, endometrial stroma	negative		negative	strong	>75%	strong
Uterus II, glandular cells	negative		negative	strong	>75%	strong
Vagina, squamous	moderate	75%-25%	moderate	strong	>75%	strong
