# Supplementary material for: Splicing factor SRSF3 represses translation of p21cip1/waf1 mRNA
Source: Cell Death Dis. 2022 Nov 7;13(11):933. doi: 10.1038/s41419-022-05371-x (PMC9640673; doi:10.1038/s41419-022-05371-x)
Supplement: Supplementary file 8 — Supplementary Table 2 [file 41419_2022_5371_MOESM8_ESM.pdf]

Supplementary Table 2 Cell microarray of p21 and SRSF3

cell line	summary_expression_value	
	p21	SRSF3
A-431	weak	strong
A-549	strong	strong
AN3-CA	negative	strong
BEWO	moderate	strong
CACO-2	negative	strong
CAPAN-2	weak	strong
Daudi	negative	strong
EFO-21	moderate	strong
HaCaT	moderate	strong
HDLM-2	weak	strong
HEK 293	negative	strong
HEL	negative	strong
HeLa	moderate	strong
Hep-G2	strong	strong
HL-60	negative	strong
HMC-1	moderate	strong
HTh 83	moderate	strong
K-562	negative	strong
Karpas-707	moderate	strong
KM3	moderate	strong
LP-1	negative	strong
MCF-7	strong	strong
MOLT-4	negative	strong
NB-4	negative	strong
NTERA-2	negative	strong
PC-3	weak	strong
RH-30	moderate	strong
RPMI-8226	negative	strong
RT-4	strong	strong
SCLC-21H	weak	strong
SH-SY5Y	moderate	strong
SiHa	moderate	strong
SK-MEL-30	weak	strong
THP-1	weak	strong
TIME	moderate	strong
U-138MG	moderate	strong
U-2 OS	moderate	strong
U-2197	negative	strong
U-251MG	moderate	strong
U-266/70	weak	strong
U-266/84	negative	strong
U-698	negative	strong
U-87MG	moderate	not representative
U-937	negative	strong
WM-115	moderate	strong

p21
Ensembl_Gene_ID: *ENSG00000124762*
Antibody_identifier (HAP-ID): *CAB000064*

SRSF3
Ensembl_Gene_ID: *ENSG00000112081*
Antibody_identifier (HAP-ID): *CAB012986*
