# Supplementary material for: Splicing factor SRSF3 represses translation of p21cip1/waf1 mRNA
Source: Cell Death Dis. 2022 Nov 7;13(11):933. doi: 10.1038/s41419-022-05371-x (PMC9640673; doi:10.1038/s41419-022-05371-x)
Supplement: Supplementary file 9 — Supplementary Table 3,4 and 5 [file 41419_2022_5371_MOESM9_ESM.pdf]

Supplementary Table 3 List of primer sequences for cloning

primer	Forward	Reverse
pCI-Flag or pGEX4T3- **SRSF3** -EcoRI/NotI	5'- AAAAGAATTCAATGCATCGTGATTCCTGTCCAT-3'	5'- AAAAGCGGCCGCCTATTTCCTTTCATTTGACCTA GATC -3'
pCI-Flag or pGEX4T3- **SRSF3_RRM** –EcoRI/NotI	5'- AAAAGAATTCAATGCATCGTGATTCCTGTCCAT-3'	5'-AAAAGCGGCCGCTCAACCATTCGACAGTTCCA CTCTCTA-3'
pCI-Flag or pGEX4T3- **SRSF3_RS** –EcoRI/NotI	5'-AAAAGAATTCAGAAAAAAGAAGTAGAAATCGTGG C-3'	5'- AAAGCGGCCGC CTATTTCCTTTCATTTGACCTAG ATC -3'
pGL3-control-**p21 cip1/waf1 5'UTR**-Luciferase HindIII / NcoI	5'AAAAAAGCTTAGCTGAGGTGTGAGCAGCTG	5'-AAAACCATGGGGCGCCTGCCGCAGAAAC-3'
pGL3-control-Luciferase- **p21 cip1/waf1 3'UTR** XbaI/XbaI	5'-AAAATCTGATCCGCCCACAGGAAGCCT-3'	5'-AAAATCTAGATTACAAGTAAAGTCACTAAGAATC A-3'

Supplementary Table 4 List of primer sequences for RNAi

siRNA	Sequence (Sense strand)
siControl	5'-GGCUACGUCCAGGAGCGCACC (dTdT)-3'
siSRSF3 #1	5'- GAGUGGAACUGUCGAAUGG (dTdT)-3'
siSRSF3 #2	5'- CGAAGUGUGUGGGUUGCUA (dTdT)-3'
siSRSF3_3'UTR	5'-GAAGUGGUGUACAGGAAAU (dTdT)-3'

Supplementary Table 5 List of primer sequences for RT-PCR and RT-qPCR

primer	Sequence (Forward/ / Reverse)
Cdkn1a (p21 cip1/waf1)	5'-GACACCACTGGAGGGTGACT-3' / 5'-CAGGTCCACATGGTCTTCCT-3'
GAPDH	5'-TGACATCAAGAAGGTGGTGA-3' / 5'-TCCACCACCCTGTTGCTGTA-3'
