# Supplementary figures and images for: Splicing factor SRSF3 represses translation of p21cip1/waf1 mRNA
Source: Cell Death Dis. 2022 Nov 7;13(11):933. doi: 10.1038/s41419-022-05371-x (PMC9640673; doi:10.1038/s41419-022-05371-x)

Figure 1D

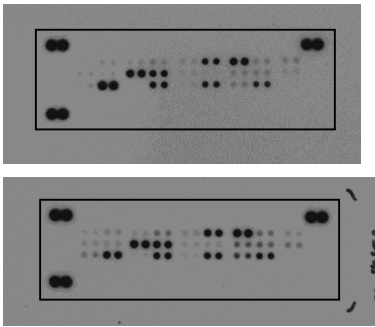

Figure 1E

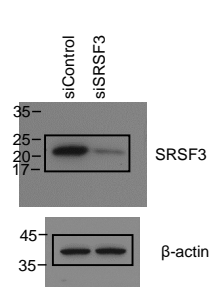

Figure 1J

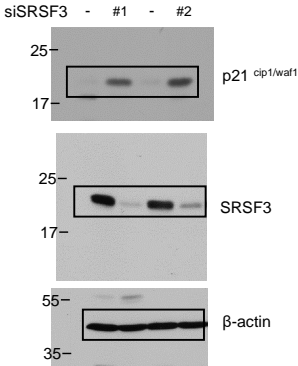

Figure 1K

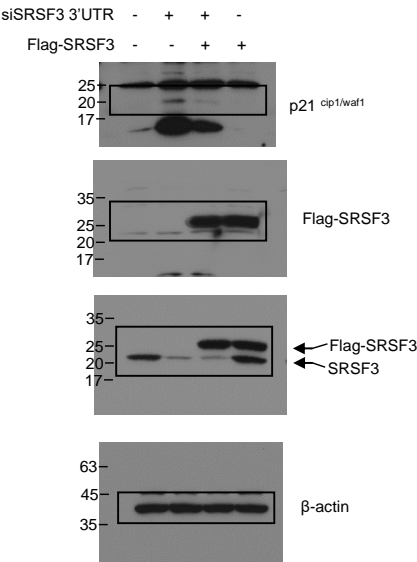

Figure 2C

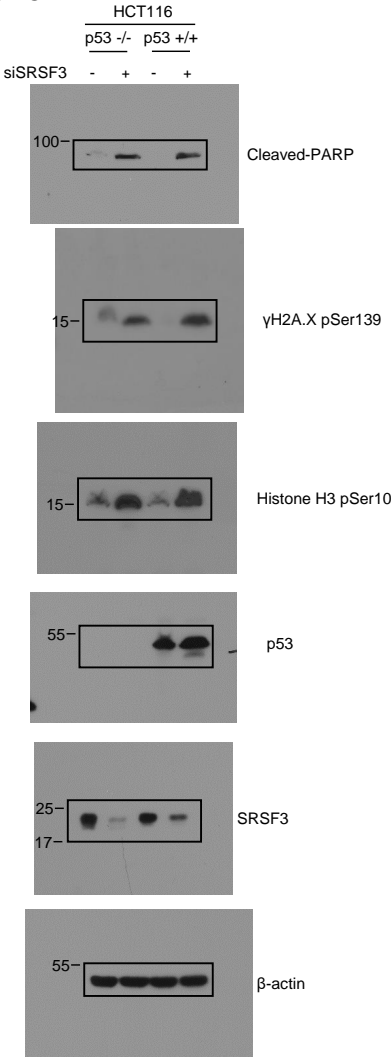

Figure 2E

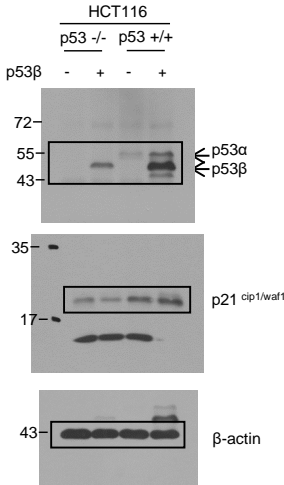

Figure 2F

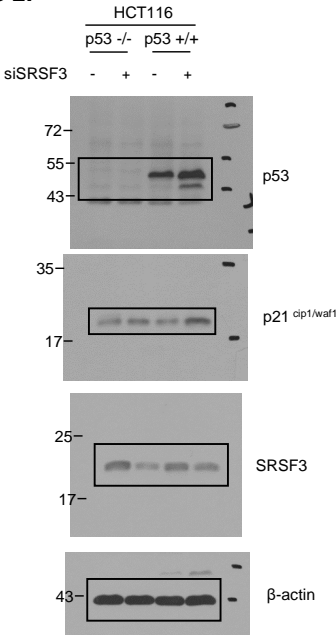

Supplement: Supplementary file 10 — Original data file_1 [file 41419_2022_5371_MOESM10_ESM.pdf]

Figure 3A

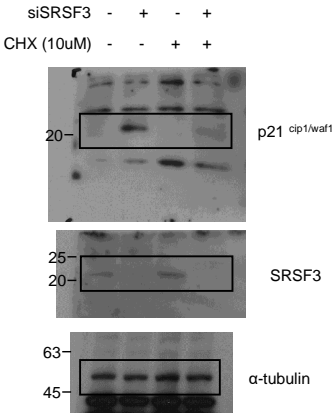

Figure 3D

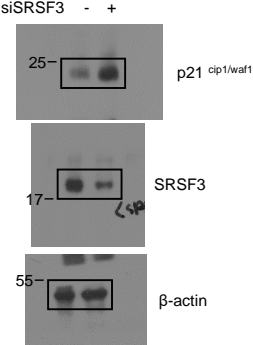

Figure 4A

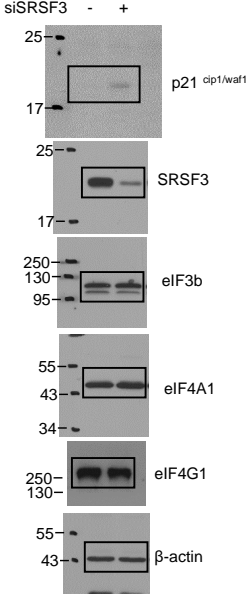

Figure 4C

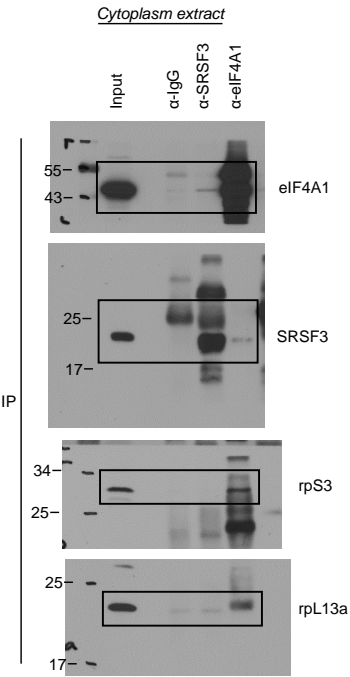

Figure 4B

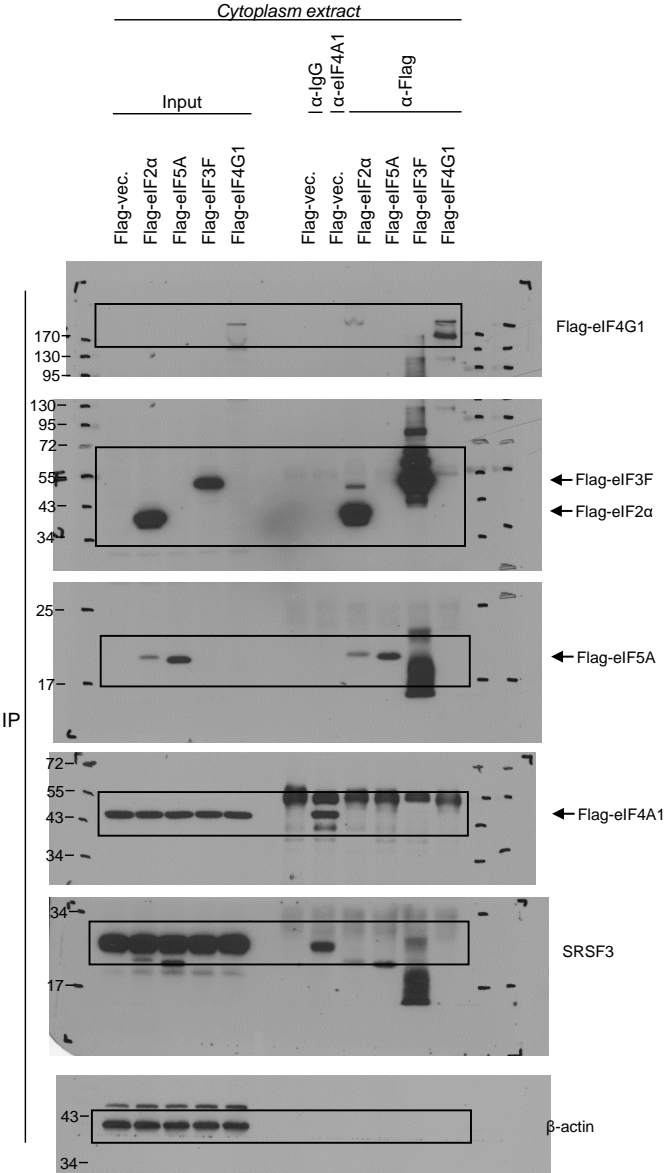

Figure 4F

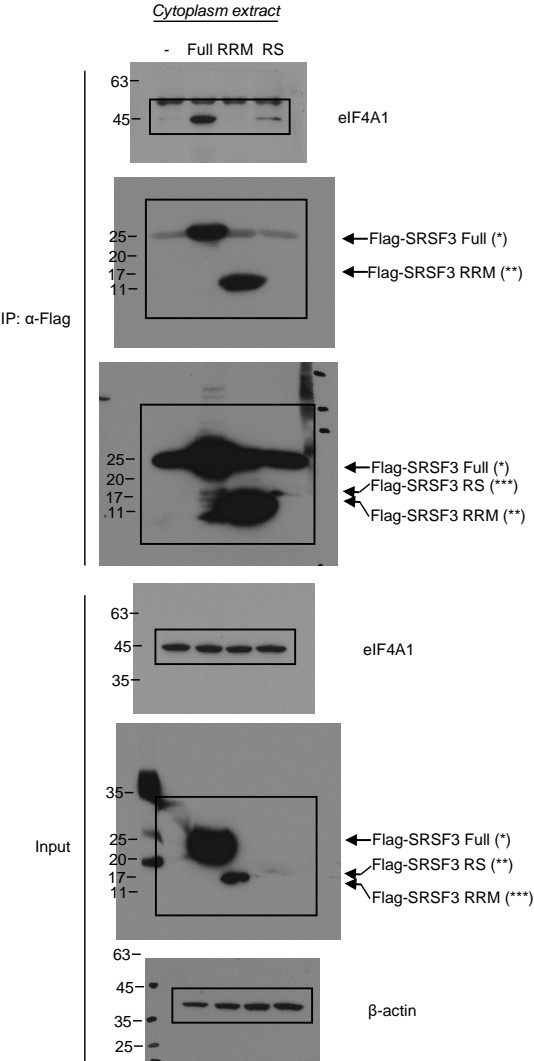

Supplement: Supplementary file 11 — Original data file_2 [file 41419_2022_5371_MOESM11_ESM.pdf]

Supplementary Figure 6

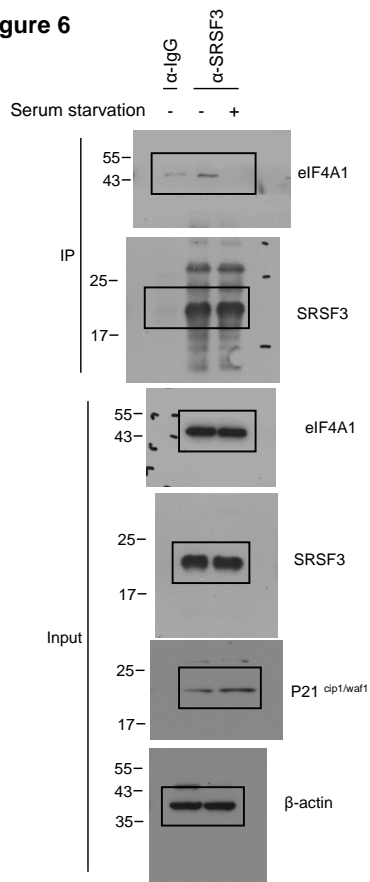

Supplement: Supplementary file 14 — Original data file_5 [file 41419_2022_5371_MOESM14_ESM.pdf]
