# Supplementary material for: Splicing factor SRSF3 represses translation of p21cip1/waf1 mRNA
Source: Cell Death Dis. 2022 Nov 7;13(11):933. doi: 10.1038/s41419-022-05371-x (PMC9640673; doi:10.1038/s41419-022-05371-x)

Original data files 3; Full blots related to Fig.4F, Fig.5 and Supplementary Fig.2

Figure 4G

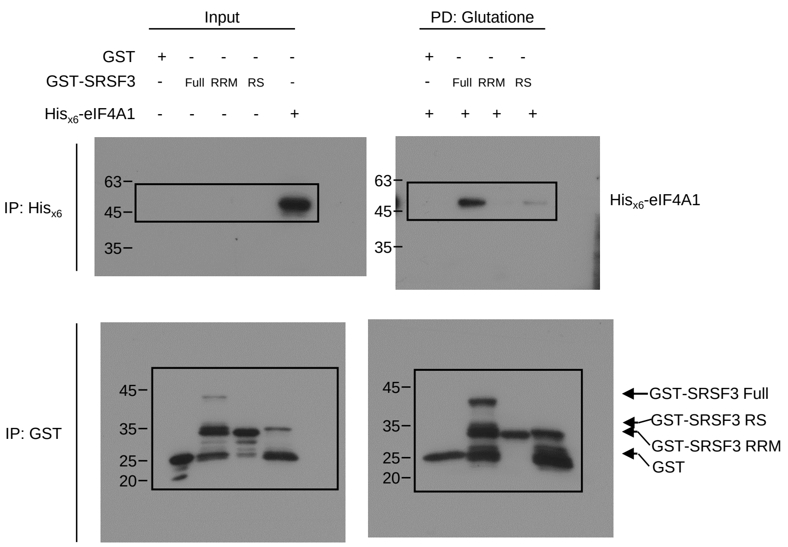

Figure 5A

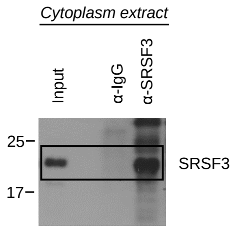

Figure 5B

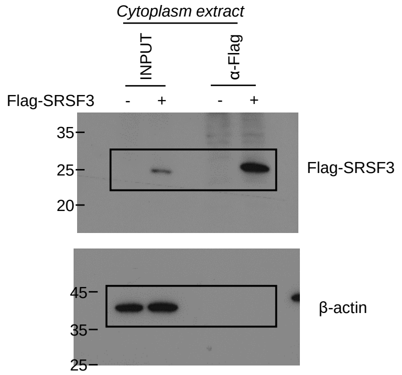

Figure 5C

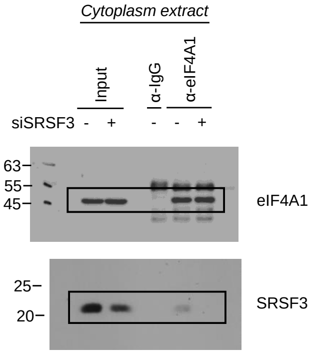

Figure 5E

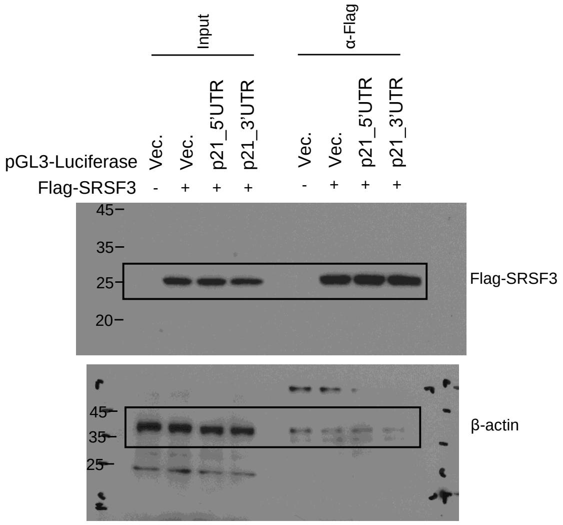

Supplementary Figure 2C

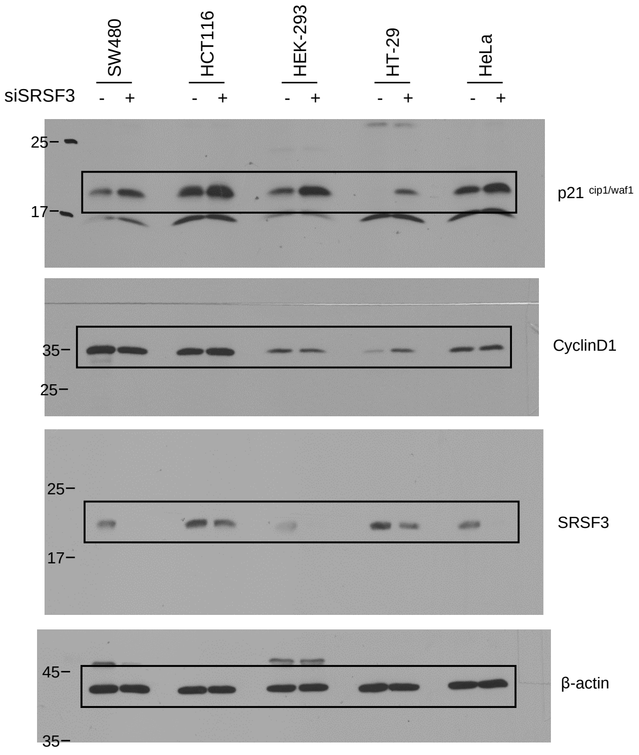

Supplementary Figure 2A

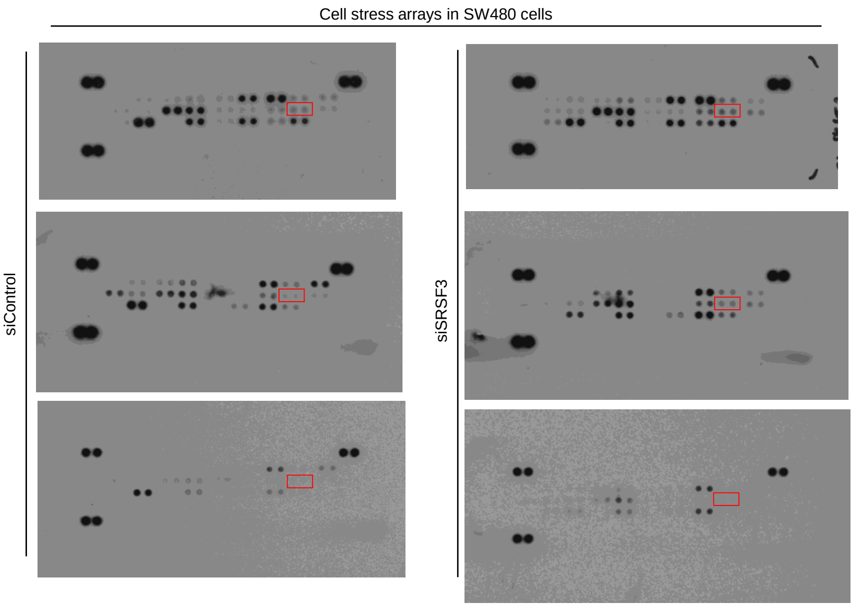

Supplement: Supplementary file 12 — Original data file_3 [file 41419_2022_5371_MOESM12_ESM.pdf]
