# Supplementary material for: Splicing factor SRSF3 represses translation of p21cip1/waf1 mRNA
Source: Cell Death Dis. 2022 Nov 7;13(11):933. doi: 10.1038/s41419-022-05371-x (PMC9640673; doi:10.1038/s41419-022-05371-x)

Original data files 4; Full blots related to Supplementary Fig.3, Supplementary Fig.4 and Supplementary Fig.5

Supplementary Figure 3A

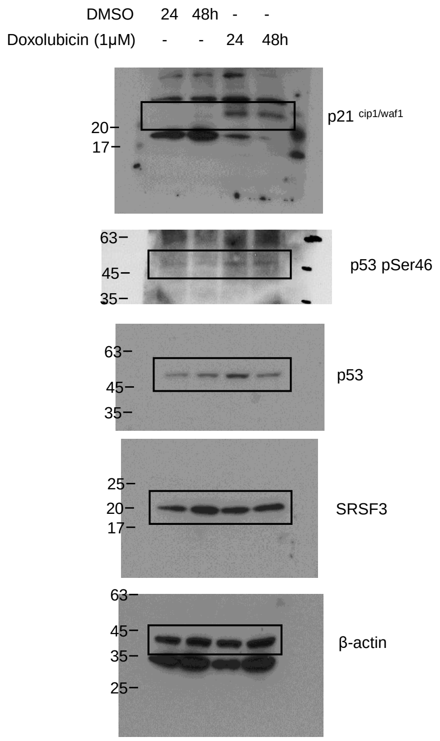

Supplementary Figure 3C

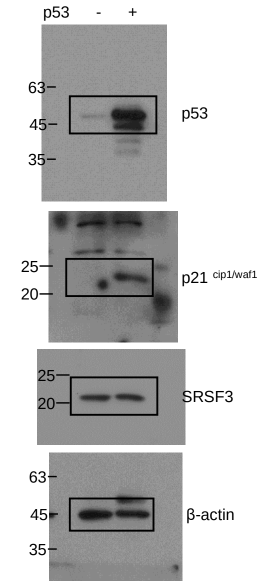

Supplementary Figure 4

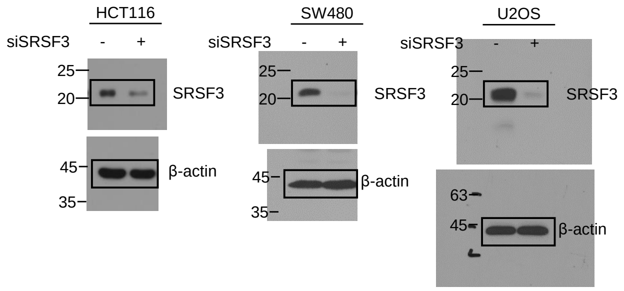

Supplementary Figure 5A

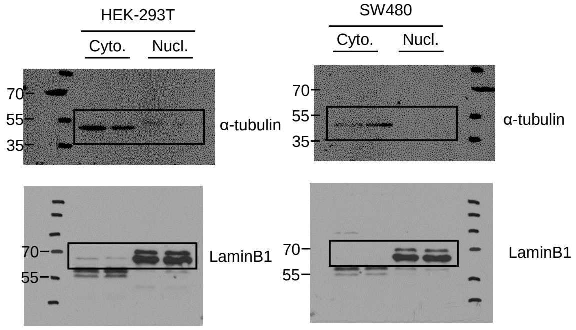

Supplementary Figure 5B

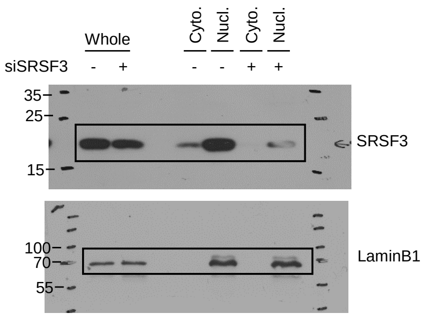

Supplement: Supplementary file 13 — Original data file_4 [file 41419_2022_5371_MOESM13_ESM.pdf]
